# Tryptophan Predicts the Risk for Future Type 2 Diabetes

**DOI:** 10.1371/journal.pone.0162192

**Published:** 2016-09-06

**Authors:** Tianlu Chen, Xiaojiao Zheng, Xiaojing Ma, Yuqian Bao, Yan Ni, Cheng Hu, Cynthia Rajani, Fengjie Huang, Aihua Zhao, Weiping Jia, Wei Jia

**Affiliations:** 1 Shanghai Key Laboratory of Diabetes Mellitus and Center for Translational Medicine, Shanghai Jiao Tong University Affiliated Sixth People's Hospital, Shanghai, China; 2 Department of Endocrinology and Metabolism, Shanghai Jiao Tong University Affiliated Sixth People’s Hospital, Shanghai Diabetes Institute, Shanghai, China; 3 University of Hawaii Cancer Center, Honolulu, United States of America; Macquarie University, AUSTRALIA

## Abstract

Recently, 5 amino acids were identified and verified as important metabolites highly associated with type 2 diabetes (T2D) development. This report aims to assess the association of tryptophan with the development of T2D and to evaluate its performance with existing amino acid markers. A total of 213 participants selected from a ten-year longitudinal Shanghai Diabetes Study (SHDS) were examined in two ways: 1) 51 subjects who developed diabetes and 162 individuals who remained metabolically healthy in 10 years; 2) the same 51 future diabetes and 23 strictly matched ones selected from the 162 healthy individuals. Baseline fasting serum tryptophan concentrations were quantitatively measured using ultra-performance liquid chromatography triple quadruple mass spectrometry. First, serum tryptophan level was found significantly higher in future T2D and was positively and independently associated with diabetes onset risk. Patients with higher tryptophan level tended to present higher degree of insulin resistance and secretion, triglyceride and blood pressure. Second, the prediction potential of tryptophan is non-inferior to the 5 existing amino acids. The predictive performance of the combined score improved after taking tryptophan into account. Our findings unveiled the potential of tryptophan as a new marker associated with diabetes risk in Chinese populations. The addition of tryptophan provided complementary value to the existing amino acid predictors.

## Introduction

Type 2 diabetes (T2D) is estimated to affect over 550 million people worldwide by 2030 [1]. T2D will further increase the risk of developing hypertension, cardiovascular disease, coronary heart disease, stroke, and several types of cancers. When T2D and/or any of the consequent metabolic diseases are diagnosed, the pathophysiological status would be extremely difficult to reverse. Therefore, the identification of future type 2 diabetes is crucial for disease prevention and early intervention.

It is commonly accepted that T2D is characterized by altered metabolic status[2, 3]. Some metabolites specific to T2D risk[4–6], diagnosis[7], and treatment assessment[8, 9] are reported in various populations. Recently, a number of studies have highlighted that the serum levels of branched-chain and aromatic amino acids, including leucine, isoleucine, valine, phenylalanine, and tyrosine, are closely correlated to insulin resistance, obesity, and future diabetes[4, 5, 10]. We also verified the T2D predictive value of these five amino acids (AAs) in Chinese populations [11].

Tryptophan, an aromatic amino acid metabolite, has expansive physiological functions in the regulation of growth and feed intake, mood and behavior, and immune responses[12, 13]. It is one of the essential amino acids for the human. However, the human body cannot synthesize it and thus it must be obtained from the diet (plants and bacteria). Our metabolic profiling study found that circulating tryptophan level rose in obese participants compared with healthy lean [14] and fell after dietary intervention[15]. Thus, the main goal of this report is to assess whether tryptophan is associated with the development of T2D. A total of 213 participants from a ten-year prospective study were involved in this work. Their baseline fasting serum tryptophan levels were quantitatively measured by a targeted ultra-performance liquid chromatography triple quadruple mass spectrometry (UPLC-TQ/MS) platform. The high risk individuals discrimination potential of tryptophan were examined in all (n = 213) and verified in strictly matched (n = 74) subjects. Its predictive performance was further compared to the other established amino acid markers.

## Materials and Methods

Written informed consent was obtained from all participants before the start of the study. The Ethics Committee of our institution approved the study, in accordance with the World Medical Association’s Declaration of Helsinki.

### Study Population

A group of 213 healthy individuals (20–75 year-old) with normal glucose tolerance was selected from a prospective cohort study called Shanghai Diabetes Study (SHDS)[16]. The SHDS started in 1997–2001, where fasting and 2 h postprandial serum and plasma of all the participants from two urban communities in Shanghai were collected and stored. After a median follow-up time of 10.0 years (SD = 3.1), 51 individuals (43% male) developed diabetes and 162 (27% male) remained free of type 2 diabetes. Twenty-three of the 162 healthy subjects were selected further with strictly matched age, BMI, cholesterol, triglyceride, glucose and insulin levels to future T2D. The diagnosis criteria of T2D was based on the 1999 WHO criteria: fasting plasma glucose ≥ 7.0mmol/l and/or 2 h plasma glucose ≥ 11.1mmol/l[17].

### Sample Collection

Serum samples were collected and stored following the standard operation protocol of Sixth People’s Hospital of Shanghai, China. Briefly, fasting venous blood samples were centrifuged immediately after collection from the subjects in the morning, and the resulting serum were delivered by dry ice storage boxes to the laboratory study and stored in aliquots in a -80°C freezer until sample preparation.

### Metabolic Markers

Fasting and 2 h postprandial plasma glucose (an OGTT test) and insulin levels (Glucose0, Glucose120, INS0, and INS120), serum lipid profiles (total cholesterol TC, triglyceride TG, high-density lipoprotein-cholesterol HDL, low-density lipoprotein-cholesterol LDL), blood pressure (systolic and diastolic blood pressure SP and DP), waist circumference, body mass index (BMI), and liver and kidney functions were determined as previously described[11]. Insulin resistance and secretion were measured by Homeostatic Model Assessment of insulin resistance (HOMA-IR = Glucose0*INS0/22.5), Matsuda index (10000/ (Glucose0×INS0×Glucose120×INS120)^0.5^), and Homeostatic Model Assessment of beta-cell function (HOMA-Beta = 20*INS0/ (Glucose0-3.5)[18, 19]. First- and second-phases of insulin secretion were estimated using the Stumvoll formula: first-phase secretion = 2032+4.681*INS0-135*Glucose120+0.995*INS120+27.99*BMI-269.1*Glucose0 and second-phase secretion = 277+0.8*INS0-42.79*Glucose120+0.321*INS120+5.338*BMI[20]. All study measures were obtained before 10 a.m. after an overnight fast in accordance as well with the standard operation protocol of the Sixth People’s Hospital of Shanghai, China.

### Quantitative Measurement of Tryptophan

The fasting serum levels of tryptophan in all the enrolled participants were analysed by UPLC-TQ/MS (Waters, Milford, MA, USA). Briefly, A 40 μL aliquot of serum sample was used in UPLC-TQ/MS ESI+ analysis. After diluted with 80 μL of water, the sample was extracted with 500 μL of a mixture of methanol and acetonitrile (1:9, v/v). The extraction procedure was performed at -20°C for 10 min after 2 min vortexing and 1 min ultrasonication. The sample was then centrifuged at 4°C at 12000 rpm for 15 min. An aliquot of 20 μL supernatant was vacuum-dried at room temperature. After that, the residue was redissolved by 100 μL of a mixture of methanol and water (1:1, v/v) with 1 μg/mL of L-2-chlorophenylalanine followed by the same vortexing, ultrasonication and centrifugation steps ahead. A volume of 80 μL supernatant was trasferred into the sampling vial for UPLC-TQ/MS analysis (Waters, Manchester, U.K.). In addition to the internal standards used for quality contol, a quality control (QC) samples consisting of five reference standards was prepared and run after each 10 serum samples. The QC samples were kept at 10°C during the entire analysis. A 5 μL aliquot of sample was injected into an ultraperformance liquid chromatography system (Waters, U.K.) with a 4.6 mm × 150 mm, 5 μmElispse XDB-C18 column (Agilent, USA). The column was held at 40°C. The elution procedure for the column was 1% for the first 0.5 min,1–20% B over 0.5–9 min, 20–75% B over 9–11 min, 75–99% B over 11–16 min, and the composition was held at 99% B for 0.5 min, where A = water with 0.1% formic acid and B = acetonitrile with 0.1% formic acid for positive mode (ESI+) and the flow rate was 0.4 mL/min. A Waters XEVO-Triple Quadrupole MS was used for the mass spectrometry detection. The temperature for the source and desolvation gas was set at 150 and 450°C respectively. The gas flow for cone and desolvation was 50 and 800 L/h respectively. The capillary voltage was set to 3.0 kV. All the compounds were detected in multiple reaction monitoring (MRM) mode.

### Statistics

The acquired raw data from UPLC-TQ/MS were initially processed by TargetLynx software (v 4.1, Waters, USA), and then corrected manually to ensure data quality. Data in tables and figures were expressed as mean ± S.E. All statistical tests were two-sided, and p values less than 0.05 were considered statistically significant. All statistical analysis and graphics were carried out using SPSS (V19, IBM, USA) and Graphpad Prism (6.0, Graphpad, USA). Raw data was provided as S1 File.

Non-parametric Mann-Whitney U test was applied for comparing the difference between two groups, as over 90% variables were deviated from normality from Kolmogorov-Smirnov normality test. Spearman rank correlation coefficients were calculated to measure the relationships between the circulating levels of tryptophan and metabolic markers. Basic and advanced logistic regression models were fitted to assess the T2D risk prediction ability. Crude odds ratio was derived from basic model with individual variable only and odds ratio was from advanced model with variable of interest and confounding factors including age, gender, BMI, fasting and 2 h postprandial glucose, fasting and 2 h postprandial insulin, and HOMA-IR. ROC analysis and AUC (area under ROC) was conducted to evaluate the discrimination performance. The 5AAs- and 6AAs- combined scores were generated by linear regression based on the abundance of 5 or 6 amino acids of interest.

## Results

### Serum Tryptophan Was Positively Associated with T2D Risk

**Fig 1:**
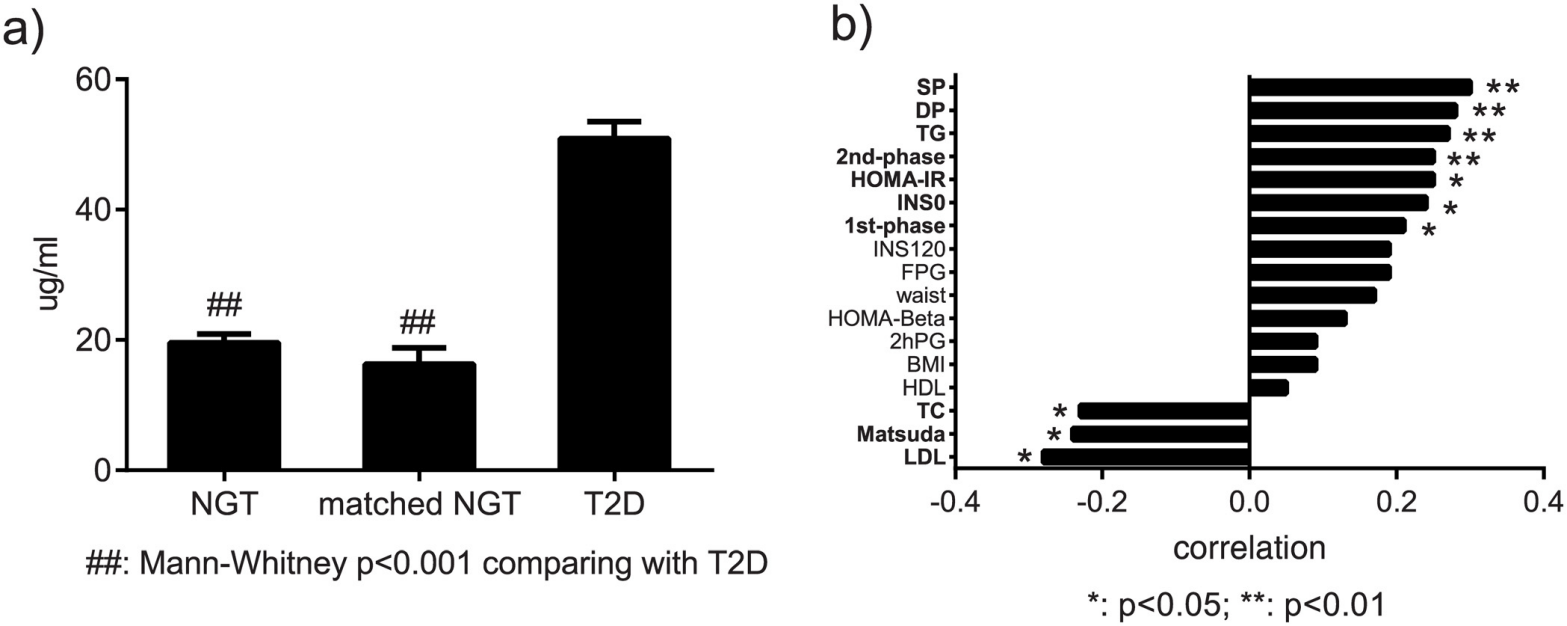
a) Baseline tryptophan levels (mean with S.E.) in future NGT (n = 162), matched future NGT (n = 23), and future T2D (n = 51) groups. b) Association of tryptophan with metabolic markers in all participants (n = 213). **Abbreviations:** BMI = body mass index; Glucose0 = fasting plasma glucose; Glucose120 = 2 h plasma glucose; INS0 = fasting insulin; INS120 = 2 h insulin; HDL = high-density lipoprotein; LDL = low-density lipoprotein; SP = systolic blood pressure; DP = diastolic blood pressure; TC = total cholesterol; TG = total triglycerides; HOMA-IR = homeostatic model assessment of insulin resistance (FPG*INS0/22.5); Matsuda index = 10000/(Glucose0×INS0×Glucose120×INS120)^0.5^; HOMA-Beta = homeostatic model assessment of beta-cell function (20*INS0/(Glucose0-3.5); First-phase secretion = 2032+4.681*INS0-135*Glucose120+0.995*INS120+27.99*BMI-269.1*Glucose0; Second-phase secretion = 277+0.8*INS0-42.79*Glucose120+0.321*INS120+5.338*BMI.

From the 213 healthy participants, 51 developed type 2 diabetes (named T2D) after 10 years while 162 remained healthy with normal glucose tolerance (named NGT) [16]. Compared to the NGT group, the T2D group had higher levels of tryptophan (Table 1). However, we noticed that the glucose, insulin, and blood pressure levels were also higher in T2D group, compared to NGT group. We thus selected 23 from the 162 healthy controls with comparative age, BMI, cholesterol, triglyceride, glucose and insulin levels to the T2D group (named matched NGT). As expected, the baseline serum tryptophan level was significantly higher in T2D, compared to matched NGT, with a fold change of 3.06 and a p value lower than 0.01 (Table 1 and Fig 1a). The ROC analysis demonstrated once again tryptophan was a good discriminator between T2D and NGT, regardless of all (AUC = 0.88) or matched (AUC = 0.92) subjects were involved. Then, logistic regression models, using all and matched subjects, were fitted and further confirmed that tryptophan was positively associated with diabetes risk and independent of both physical and metabolic markers including age, gender, BMI, fasting and 2 h postprandial glucose, fasting and 2 h postprandial insulin, and HOMA-IR (p trend < 0.001 for both; odds ratio of all participants = 2.54 (95%CI: 1.85, 3.48); odds ratio of matched subjects = 5.06 (95%CI: 2.31, 11.10)). Additionally, correlation analysis involving all the 213 subjects demonstrated that tryptophan was positively correlated with TG, 1^st^- and 2^nd^ phase beta cell secretion, fasting insulin, HOMA-IR, and was negatively correlated with LDL, Matsuda index and TC (Fig 1b).

**Table 1:** Baseline clinical characteristics and tryptophan levels in individuals who developed diabetes (T2D, n = 51) and remained metabolically healthy (NGT, n = 162) in 10 years, and in matched future metabolically healthy (matched NGT, n = 23) individuals.

Metabolic markers	NGT (n = 162)	Matched NGT (n = 23)	T2D (n = 51)
Gender (male:female)	44:118	11:12	24:27
Age (yrs)	38.51±0.95^##^	52.7±2.72	53.78±1.75
BMI (kg/m2)	24.63±0.27	25.75±0.62	25.53±0.50
Waist (cm)	76.91±0.71^#^	80.13±1.98	80.12±1.13
Glucose0 (mM)	4.76±0.04^##^	4.95±0.08	5.13±0.09
Glucose120 (mM)	5.21±0.09^##^	6.07±0.24	6.08±0.18
INS0 (U/L)	7.90±0.40^##^	9.89±1.13	9.36±0.56
INS120 (U/L)	45.15±3.46^##^	52.66±7.79	56.42±4.20
TC (mM)	4.05±0.03	4.14±0.1	3.97±0.02
TG (mM)	1.05±0.02^##^	1.23±0.06	1.33±0.06
HDL (mM)	1.36±0.01	1.38±0.03	1.33±0.02
LDL (mM)	2.66±0.03^##^	2.73±0.07	2.88±0.10
SP (mmHg)	112.16±0.93^##^	119.26±2.23	122.51±1.51
DP (mmHg)	73.41±0.54^##^	75.78±1.13	79.14±1.13
HbA1c (%)	5.70±0.01^##^	5.71±0.05	5.80±0.07
HOMA-IR	1.66±0.08^##^	2.22±0.27	2.14±0.13
HOMA-Beta	148.83±12.37	137.54±14.64	138.68±13.53
Matsuda index	176.60±11.68^##^	116.44±15.37	93.68±6.33
First-phase secretion	828.06±40.76^##^	700.14±38.21	645.08±60.8
Second-phase secretion	208.4±9.12^##^	179.36±8.94	178.88±12.54
Tryptophan (ug/ml)	19.58±1.36^##^	16.64±2.14^##^	50.93±2.61

Data present as mean ± S.E.

^#^ Mann-Whitney p < 0.05 compared with T2D.

^##^ Mann-Whitney p < 0.01 compared with T2D.

**Abbreviations:** BMI = body mass index; Glucose0 = fasting plasma glucose; Glucose120 = 2 h plasma glucose; INS0 = fasting insulin; INS120 = 2 h insulin; HDL = high-density lipoprotein; LDL = low-density lipoprotein; SP = systolic blood pressure; DP = diastolic blood pressure; TC = total cholesterol; TG = total triglycerides; HOMA-IR = homeostatic model assessment of insulin resistance (FPG*INS0/22.5); Matsuda index = 10000/(Glucose0×INS0×Glucose120×INS120)^0.5^; HOMA-Beta = homeostatic model assessment of beta-cell function (20*INS0/(Glucose0-3.5); First-phase secretion = 2032+4.681*INS0-135*Glucose120+0.995*INS120+27.99*BMI-269.1*Glucose0; Second-phase secretion = 277+0.8*INS0-42.79*Glucose120+0.321*INS120+5.338*BMI.

### Predictive Value Adds to Existing Markers

**Fig 2:**
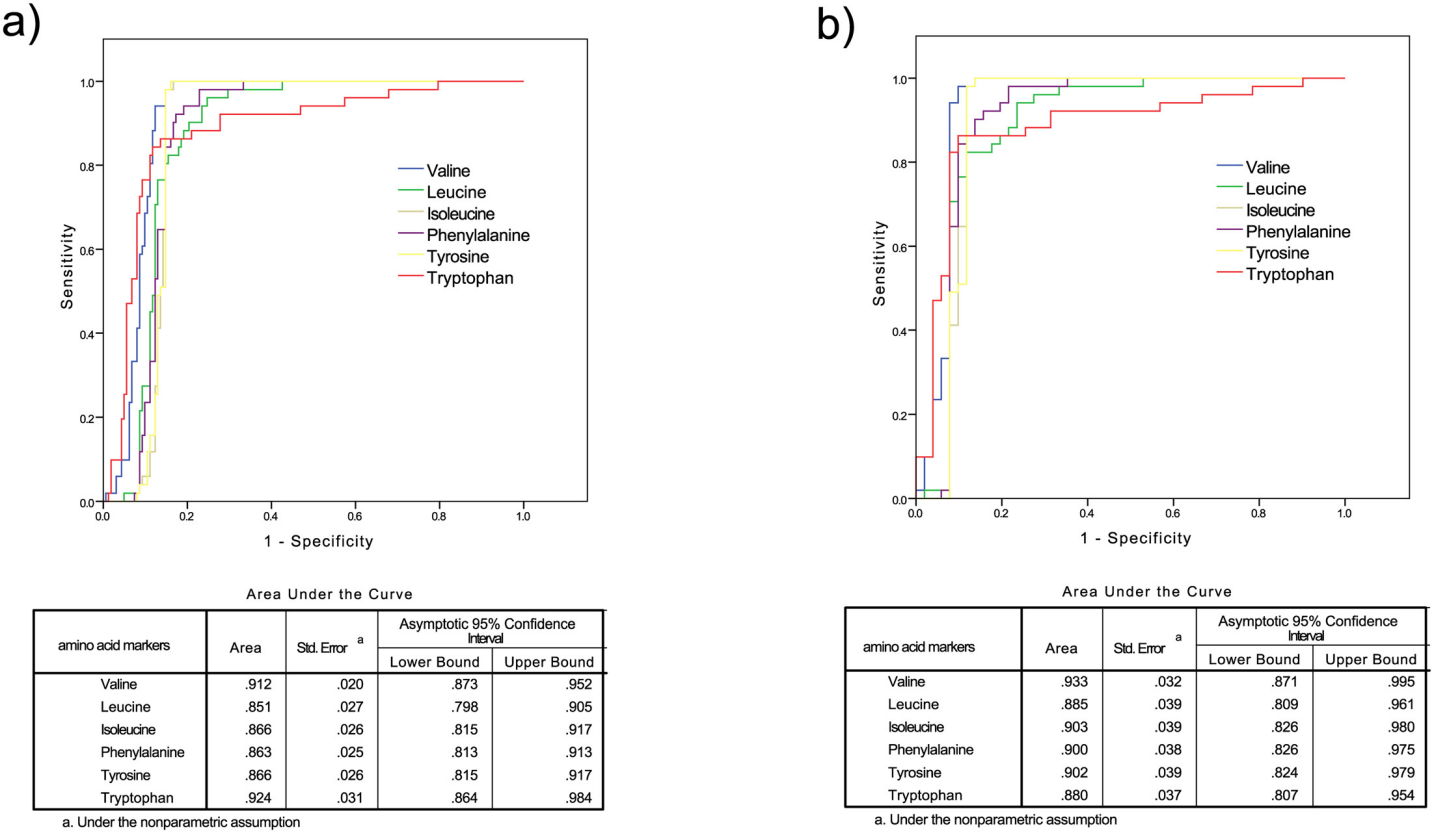
ROCs of 5AAs-combined score and 6-AAs combined score in discriminating a) T2D and matched NGT (n = 74); and b) T2D and NGT (n = 213).

First, we compared the predictive and discriminant performances of tryptophan with the other 5 AAs (valine, leucine, isoleucine, phenylalanine, and tyrosine) which were recently reported as new biomarkers for T2D risk. All the individual amino acids were significantly higher in future T2D with a fold change (T2D/NGT) range of 2.01–3.06 (Table 2 for matched and Table 3 for all participants). Similarly, basic logistic regression models indicated that their p trend values were all lower than 0.002 and the crude odds ratios ranged from 1.42 to 4.10 (Tables 2 and 3). Once again, as Fig 2 illustrated, tryptophan (the red line) was non-inferior to the 5 AAs in discriminating future T2D and future NGT (with comparable AUCs). Second, we further assessed the overall performance of the combined score with and without tryptophan. The 6AAs-combined score, derived from tryptophan and the 5 AAs, overperformed all the individual AAs and the 5AAs-combined score, with the fold changes (T2D/NGT) of 3.33 and 3.73, AUCs of 0.95 and 1.00, and crude odds ratios of 3.88 (2.11, 7.13) and 3.50(1.85, 6.60), in matched and all subjects respectively (Tables 2 and 3). Taken together, tryptophan held comparable and complementary value to the established AA predictors.

**Table 2:** Predictive performance of baseline amino acids and combined scores in discriminating individuals who developed diabetes in 10 years (T2D, n = 51) from those who remained metabolically healthy (matched NGT, n = 23).

Amino acids and combined scores	p1	FC	Basic logistic model	
			Crude OR (95% CI)	p2
Valine	<0.001	2.63	2.99 (1.86, 4.81)	<0.001
Leucine	<0.01	2.07	2.65 (1.45, 4.84)	0.002
Isoleucine	<0.001	2.76	4.10 (2.12, 7.93)	<0.001
Phenylalanine	<0.01	2.07	3.22 (1.62, 6.41)	0.001
Tyrosine	<0.001	2.31	3.89 (2.04, 7.42)	<0.001
Tryptophan	<0.001	3.06	3.74 (2.12, 6.58)	<0.001
5AAs-Combined score	<0.001	3.20	3.58 (1.98, 6.48)	<0.001
6AAs-Combined score	<0.001	3.33	3.88 (2.11, 7.13)	<0.001

**Abbreviations:** 5AAs-Combined score: the combined score derived from the abundance of 5 AAs (valine, leucine, isoleucine, tyrosine, and phenelalanine); 6AAs-Combined score: the combined score derived from the abundance of 6 AAs (valine, leucine, isoleucine, tyrosine, phenelalanine, and tryptophan).

p1 was the p value from Mann Whitney U test compared T2D and NGT.

FC (fold change) represents the mean ratio of T2D to NGT.

Crude odds ratio (Crude OR) and confidence interval (CI) per s.d., and p2 were from basic logistic regression models and S.D. scaled data.

**Table 3:** Predictive performance of baseline amino acids and combined scores in discriminating individuals who developed diabetes in 10 years (T2D, n = 51) from those who remained metabolically healthy (NGT, n = 162).

Amino acids and combined scores	p1	FC	Basic logistic model	
			Crude OR (95% CI)	p2
Valine	<0.001	2.52	1.83 (1.51, 2.23)	<0.001
Leucine	<0.001	2.06	1.94 (1.37, 2.76)	<0.001
Isoleucine	<0.001	2.60	1.50 (1.27, 1.83)	<0.001
Phenylalanine	<0.001	2.01	1.42 (1.18, 1.71)	<0.001
Tyrosine	<0.001	2.28	1.50 (1.24, 1.81)	<0.001
Tryptophan	<0.001	2.60	2.00 (1.65, 2.41)	<0.001
5AAs-Combined score	<0.001	2.72	1.47 (1.25, 1.75)	<0.001
6AAs-Combined score	<0.001	3.73	3.50(1.85, 6.60)	<0.001

**Abbreviations:** 5AAs-Combined score: the combined score derived from the abundance of 5 AAs (valine, leucine, isoleucine, tyrosine, and phenylalanine); 6AAs-Combined score: the combined score derived from the abundance of 6 AAs (valine, leucine, isoleucine, tyrosine, phenylalanine, and tryptophan).

p1 was the p value from Mann Whitney U test compared T2D and NGT.

FC (fold change) represents the mean ratio of T2D to NGT.

Crude odds ratio (Crude OR) and confidence interval (CI) per s.d., and p2 were from basic logistic regression models and S.D. scaled data.

## Discussion

The strong association of branched-chain and aromatic amino acids with diabetes risk were reported and validated in various populations although the mechanisms underlying these observations are remain unclear [4, 7, 10, 21]. The primary focus of this study was to assess the predictive power of serum tryptophan level in response to the occurrence of type 2 diabetes. We provided the first evidence that, serum tryptophan level was positively and independently associated with diabetes development risk in view of all and matched individuals. These findings, which highlight the ability of tryptophan to identify high risk individuals before the onset of T2D, and even before significant variations of metabolic markers, are noteworthy. The implementation of preventive therapies on high risk individuals is possible considering tryptophan level, along with other metabolites and/or clinical markers associated with T2D risk. Additionally, as a routine constituent of most protein-based foods or dietary proteins, serum tryptophan level could be regulated conveniently by diet, as long as the metabolic and physiological mechanism and safety is clarified.

Tryptophan metabolism has been reported highly associated with insulin resistance and diabetes risk[12, 22]. The activity of rate-limiting enzyme of tryptophan-kynurenine, indoleamine-2,3-dioxygenase (IDO), was enhanced significantly in T2D patients[23], thus downstream metabolites such as kynurenine, kynurenic acid, xanthurenic acid and hydroxykynurenine, were higher in T2D than in non-diabetic subjects[24, 25], although inconsistent observations of tryptophan levels were reported[25, 26]. Different from previous reports, our study focused on the predictive value of baseline tryptophan for future T2D which might contribute to early screening and intervention in clinic. Interestingly, our recent gastric bypass surgery study[27] (n = 33) showed that baseline serum tryptophan level was negatively associated with diabetes duration (S1 Fig). Adams et al.[28] reported that plasma levels of valine and leucine were elevated gradually during diabetes development in the UCD-T2D (University of California at Davis Type 2 Diabetes Mellitus) rat model. Taken together, it might be supposed that, serum tryptophan levels increased before insulin resistance and T2D and then depleted gradually along with the progression of T2D. The variation pattern of circulating tryptophan may represent the compensatory metabolic response to increased oxidative stress related to inflammation as well as the competition with branched-chain amino acids for the same trans-membrane transporter during the development of T2D.

It is a consensus that T2D begins with insulin resistance of peripheral tissues [29]. To compensate for this resistance, pancreatic beta-cells respond with increased insulin synthesis and proliferation[30]. The longer the duration, the more severe the condition is. It is estimated by United Kingdom Prospective Diabetes Study (UKPDS) that only 50% of normal beta-cell function remains at the onset of diabetes[31]. In the present study, as association analysis indicated, patients with higher tryptophan level tended to present higher degree of insulin resistance (HOMA-IR and Matsuda index), and more active insulin secretion (1^st^- and 2^nd^- phase secretion). These results were in line with previous findings that tryptophan [32] and its downstream metabolite serotonin (5-hydroxytryptamine) [33, 34], kynurenine [35] and xanthurenic acid [24] play key roles in the regulation of insulin resistance, pancreatic beta-cell function, and glucose level homeostasis.

Accumulating evidences suggest that intestinal microbiota composition and perturbation represent a very important environmental factor to the development and treatment of type 2 diabetes, by influencing bile acid metabolism, glucose and lipid metabolism, proinflammatory activity, and insulin resistance[36, 37]. One of the key physiological functions of tryptophan is to serve as nitrogen source for the growth of some microbes (e.g. *Escherichia coli* and *Klebsiella pneumoniae*)[38], and therefore, the alteration of serum tryptophan level is indicative of gut microbiota fermentation. In this report, baseline tryptophan level was different in future T2D and future NGT (Fig 1a and Table 1), supporting previous metagenomic findings that patients with impaired glucose metabolism, insulin resistance, or T2D were featured by imbalanced microbiota, such as less butyrate-producing bacteria (*Roseburia* species and *Faecalibacterium prausnitzii*)[39, 40], elevated *proteobacteria*[41], and lower gut bacteria gene richness [42], compared with healthy subjects. Therefore, the variation of tryptophan and related microbiota, together with other internal and external risk factors, may be triggers or early markers of T2D. These evidences validated the special role of microbial ecology in diabetes treatment, including the modification of gut hormone secretion, and the changes in intestinal gluconeogenesis [43, 44].

It is interesting that individual amino acids performed differently among various populations. Phenylalanine and valine were superior to the other 3 in the studies with American populations[4], whereas tyrosine showed better performance among participants in South Asian[45]. In our previous study with Chinese population, valine was the best among the 5 AAs [11]. In this study, we found that tryptophan is non-inferior to the 5 AAs in T2D prediction and the addition of tryptophan to the combined score increased the predictive power. It is widely accepted that most diabetic patients in China had lower BMI, more abdominal fat[46], earlier beta-cell dysfunction[47] and specific genetic loci[48]. Thus, our new findings may provide additional insights in the high prevalence, aetiological mechanisms, and potential prevention and treatment targets for diabetes in Chinese populations.

The concentrations of circulating tryptophan were influenced by several amino acids (notably leucine, valine, isoleucine, tyrosine, phenylalanine and methionine) as they share the same trans-membrane protein [49]. Therefore, we assessed the predictive value of the ratios of tryptophan to the 5 amino acids (S1 Table), in addition to the 6AAs-combined score. Two of the 5 ratios, Tryptophan/Isoleucine and Tryptophan/Tyrosine, were significantly lower in future T2D compared to NGT and were associated with the risk of developing T2D, implicating that tryptophan might be suppressed by isoleucine and tyrosine in the competition. This interesting point deserves further investigation.

Our study has several limitations, such as a medium population size (especially size of the matched NGT group) due to the strict criterion for samples selection, and the short of tryptophan level after diabetes onset. The preliminary results await validation in larger and more populations.

## Supporting Information

S1 Fig Scatter plot of T2D duration (year) and tryptophan level (ug/ml). The r and p values were from Spearman correlation (n = 33).
(DOCX)

S1 File Data. (XLS)

S1 Table Predictive performance of baseline amino acid ratios in discriminating individuals who developed diabetes in 10 years (T2D, n = 51) from those who remained metabolically healthy (matched NGT, n = 23). p1 was p values from Mann Whitney U test comparing T2D and NGT. FC (fold change) represents mean ratio of T2D to NGT. Crude odds ratio (Crude OR) and confidence interval (CI) per s.d., and p2 were from basic logistic regression models and S.D. scaled data.
(DOCX)

## References

[1] Whiting DR, Guariguata L, Weil C, Shaw J. IDF diabetes atlas: global estimates of the prevalence of diabetes for 2011 and 2030. Diabetes Res Clin Pr. 2011;94(3):311–21. 10.1016/j.diabres.2011.10.029 22079683

[2] Bain JR, Stevens RD, Wenner BR, Ilkayeva O, Muoio DM, Newgard CB. Metabolomics applied to diabetes research: moving from information to knowledge. Diabetes. 2009;58(11):2429–43. 10.2337/db09-0580 19875619 PMC2768174

[3] Menni C, Fauman E, Erte I, Perry JR, Kastenmüller G, Shin SY, et al Biomarkers for type 2 diabetes and impaired fasting glucose using a nontargeted metabolomics approach. Diabetes. 2013;62(12):4270–6. 10.2337/db13-0570 23884885 PMC3837024

[4] Wang TJ, Larson MG, Vasan RS, Cheng S, Rhee EP, McCabe E, et al Metabolite profiles and the risk of developing diabetes. Nat Med. 2011;17(4):448–54. 10.1038/nm.2307 21423183 PMC3126616

[5] Weurtz P, Soininen P, Kangas AJ, Ronnemaa T, Lehtimaki T, Kahonen M, et al Branched-Chain and Aromatic Amino Acids Are Predictors of Insulin Resistance in Young Adults. Diabetes Care. 2013;36:648–55. 10.2337/dc12-0895 23129134 PMC3579331

[6] Mahendran Y, Cederberg H, Vangipurapu J, Kangas AJ, Soininen P, Kuusisto J, et al Glycerol and fatty acids in serum predict the development of hyperglycemia and type 2 diabetes in Finnish men. Diabetes Care. 2013;36(11):3732–8. 10.2337/dc13-0800 24026559 PMC3816902

[7] Tisnnrn M, Makinen VP, Kangas AJ, Soinnen P, Saltevo J, Kiukaanniemi SK, et al Circulating Metabolite Predictors of Glycemia in Middle-Aged Men and Women. Diabetes Care. 2012;35:1749–56. 10.2337/dc11-1838 22563043 PMC3402262

[8] Bao Y, Zhao T, Wang X, Qiu Y, Su M, Jia W, et al Metabonomic Variations in the Drug-Treated Type 2 Diabetes Mellitus Patients and Healthy Volunteers. J Proteome Res. 2009;(8):1623–30. 19714868 10.1021/pr800643w

[9] Zhang M, Luo H, Xi Z, Rogaeva E. Drug repositioning for diabetes based on 'omics' data mining. PloS one. 2015;10(5):e0126082 10.1371/journal.pone.0126082 25946000 PMC4422696

[10] Batch BC, Shah SH, Newgard CB, Turer CB, Haynes C, Bain JR, et al Branched chain amino acids are novel biomarkers for discrimination of metabolic wellness. Metabolism. 2013;62:961–9. 10.1016/j.metabol.2013.01.007 23375209 PMC3691289

[11] Chen T, Ni Y, Ma X, Bao Y, Liu J, Huang F, et al Branched-chain and aromatic amino acid profiles and diabetes risk in Chinese populations. Sci Rep. 2016. Epub 2016. 10.1038/srep20594 PMC4742847 26846565

[12] Floc'h NL, Otten W, Merlot E. Tryptophan metabolism, from nutrition to potential therapeutic applications. Amino Acids. 2011;(41):1195–205. 10.1007/s00726-010-0752-7 20872026

[13] Iida H, Ogihara T, Min M-k, Hara A, Kim YG, Fujimaki K, et al Expression mechanism of tryptophan hydroxylase 1 in mouse islets during pregnancy. J Mol Endocrinol. 2015;55(1):41–53. 10.1530/JME-14-0299 26136513

[14] Xie G, Ma X, Zhao A, Wang C, Zhang Y, Nieman D, et al The Metabolite Profiles of the Obese Population Are Gender-Dependent. J Proteome Res. 2014;13(9):4062–73. 10.1021/pr500434s 25132568 PMC6098236

[15] Gu Y, Zhao A, Huang F, Zhang Y, Liu J, Wang C, et al Very Low Carbohydrate Diet Significantly Alters the Serum Metabolic Profiles in Obese Subjects. J Proteome Res. 2013;(12):5801–11. 10.1021/pr4008199 24224694 PMC6088239

[16] Jia WP, Pang C, Chen L, Bao YQ, Lu JX, Lu HJ, et al Epidemiological characteristics of diabetes mellitus and impaired glucose regulation in a Chinese adult population: the Shanghai Diabetes Studies, a cross-sectional 3-year follow-up study in Shanghai urban communities. Diabetologia. 2007;50(2):286–92. 17180353 10.1007/s00125-006-0503-1

[17] World Health Organization DoNDSG. Definition, Diagnosis and Classification of Diabetes Mellitus and its Complications 1999. 1999.

[18] Matthews D, Hosker J, Rudenski A, Naylor B, Treacher D, Turner R. Homeostasis model assessment: insulin resistance and beta-cell function from fasting plasma glucose and insulin concentrations in man. Diabetologia. 1985;28(7):412–9. 3899825 10.1007/BF00280883

[19] Katz A, Nambi SS, Mather K, Baron AD, Follmann DA, Sullivan G, et al Quantitative insulin sensitivity check index: a simple, accurate method for assessing insulin sensitivity in humans. J Clin Endocr Metab. 2000;85(7):2402–10. 10902785 10.1210/jcem.85.7.6661

[20] Stumvoll M, Haeften TV, Fritsche A, Gerich J. Oral glucose tolerance test indexes for insulin sensitivity and secretion based on various availabilities of sampling tim. Diabetes Care. 2001;24(4):796–7. 11315860 10.2337/diacare.24.4.796

[21] Newgard CB. Interplay between lipids and branched-chain amino acids in development of insulin resistance. Cell Metab. 2012;15:606–14. 10.1016/j.cmet.2012.01.024 22560213 PMC3695706

[22] Oxenkrug G. Insulin Resistance and Dysregulation of Tryptophan–Kynurenine and Kynurenine–Nicotinamide Adenine Dinucleotide Metabolic Pathways. Mol Neurobiol. 2013;48(2):294–301. 10.1007/s12035-013-8497-4 23813101 PMC3779535

[23] Oxenkrug G, van der Hart M, Summergrad P. Elevated anthranilic acid plasma concentrations in type 1 but not type 2 diabetes mellitus. Integrative molecular medicine. 2015;2(5):365–8. 10.15761/IMM.1000169 26523229 PMC4624227

[24] Reginaldo C, Jacques P, Scott T, Oxenkrug G, Selhub J, Paul L. Xanthurenic acid is associated with higher insulin resistance and higher odds of diabetes. The FASEB Journal. 2015;29(1(Supplement 919.20)).

[25] Oxenkrug GF. Increased Plasma Levels of Xanthurenic and Kynurenic Acids in Type 2 Diabetes. Mol Neurobiol. 2015;52(2):805–10. 10.1007/s12035-015-9232-0 26055228 PMC4558247

[26] Herrera R, Manjarrez G, Nishimura E, Hernandez J. Serotonin-related tryptophan in children with insulin-dependent diabetes. Pediatr Neurol. 2003;28(1):20–3. . 12657415 10.1016/s0887-8994(02)00462-9

[27] Yu H, Ni Y, Bao Y, Zhang P, Zhao A, Chen T, et al Chenodeoxycholic Acid as a Potential Prognostic Marker for Roux-en-Y Gastric Bypass in Chinese Obese Patients. J Clin Endocr Metab. 2015. Epub September 24, 2015. 10.1210/jc.2015-2884 26425885

[28] Piccolo BD, Graham JL, Stanhope KL, Fiehn O, Havel PJ, Adams SH. Plasma amino acid and metabolite signatures tracking diabetes progression in the UCD-T2DM rat model. American journal of physiology Endocrinology and metabolism. 2016;310(11):E958–69. 10.1152/ajpendo.00052.2016 . 27094034 PMC4935135

[29] Samuel VT, Shulman GI. Mechanisms for insulin resistance: common threads and missing links. Cell. 2012;(148):852–71. 22385956 10.1016/j.cell.2012.02.017 PMC3294420

[30] Lin Z, Zhou J, Li X, Song L, Hou X, Tang J, et al High-normal 2 h glucose is associated with defects of insulin secretion and predispose to diabetes in Chinese adults. Endocrine. 2015;(48):179–86. 24711220 10.1007/s12020-014-0244-8

[31] Holman RR. Assessing the potential for alpha-glucosidase inhibitors in prediabetic states. Diabetes Res Clin Pract. 1998;40 (Suppl):S21–S5. 9740498 10.1016/s0168-8227(98)00038-2

[32] Inubushi T, Kamemura N, Oda M, Sakurai J, Nakaya Y, Harada N, et al L-tryptophan suppresses rise in blood glucose and preserves insulin secretion in type-2 diabetes mellitus rats. J Nutr Sci Vitaminol (Tokyo). 2012;58(6):415–22. . 23419400 10.3177/jnsv.58.415

[33] Kim K, Oh CM, Ohara-Imaizumi M, Park S, Namkung J, Yadav VK, et al Functional role of serotonin in insulin secretion in a diet-induced insulin-resistant state. Endocrinology. 2015;156(2). 10.1210/en.2014-1687 PMC4298319 25426873

[34] Bennet H, Balhuizen A, Medina A., DekkerNitert M., OttossonLaakso E., Essén S., et al Altered serotonin (5-HT) 1D and 2A receptor expression may contribute to defective insulin and glucagon secretion in human type 2 diabetes. Peptide. 2015;71:113–20. 10.1016/j.peptides.2015.07.008 26206285

[35] Pedersen ER, Tuseth N, Eussen SJ, Ueland PM, Strand E, Svingen GF, et al Associations of plasma kynurenines with risk of acute myocardial infarction in patients with stable angina pectoris. Arteriosclerosis, thrombosis, and vascular biology. 2015;35(2):455–62. 10.1161/ATVBAHA.114.304674 . 25524770

[36] Han JL, Lin HL. Intestinal microbiota and type 2 diabetes: from mechanism insights to therapeutic perspective. World J Gastroentero. 2014;20(47). 10.3748/wjg.v20.i47.17737 PMC4273124 25548472

[37] Allin KH, Nielsen T, Pedersen O. Mechanisms in endocrinology: Gut microbiota in patients with type 2 diabetes mellitus. Eur J Endocrinol. 2015;172(4):R167–77. 10.1530/EJE-14-0874 25416725

[38] Tsavkelova E, Oeser B, Oren-Young L, Israeli M, Sasson Y, Tudzynski B, et al Identification and functional characterization of indole-3-acetamide-mediated IAA biosynthesis in plant-associated Fusarium species. Fungal Genet Biol. 2012;(49):48–57. 22079545 10.1016/j.fgb.2011.10.005

[39] Qin J, Li Y, Cai Z, Li S, Zhu J, Zhang F, et al A metagenome-wide association study of gut microbiota in type 2 diabetes. Nature. 2012;(490):55–60. 10.1038/nature11450 23023125

[40] Karlsson FH, Tremaroli V, Nookaew I, Bergström G, Behre CJ, Fagerberg B, et al Gut metagenome in European women with normal, impaired and diabetic glucose control. Nature. 2013;(498):99–103. 10.1038/nature12198 23719380

[41] Larsen N, Vogensen FK, vdBerg FWJ, Nielsen DS, Andreasen AS, Pedersen BK, et al Gut Microbiota in Human Adults with Type 2 Diabetes Differs from Non-Diabetic Adults. PloS one. 2010;5(2):e9085 10.1371/journal.pone.0009085 20140211 PMC2816710

[42] Chatelier EL, Nielsen T, Qin J, Prifti E, Hildebrand F, Falony G, et al Richness of human gut microbiome correlates with metabolic markers. Nature. 2013;(500):541–6. 10.1038/nature12506 23985870

[43] Cani PD, Osto M, Geurts L, Everard A. Involvement of gut microbiota in the development of low-grade inflammation and type 2 diabetes associated with obesity. Gut Microbes. 2012;3(4):279–88. 22572877 10.4161/gmic.19625 PMC3463487

[44] Isbell JM, RAT RA, ENH EN, JS J, JPD JP, SEP SE, et al The importance of caloric restriction in the early improvements in insulin sensitivity after Roux-en-Y gastric bypass surgery. Diabetes Care. 2010;33(7):1438–42. 10.2337/dc09-2107 20368410 PMC2890335

[45] Tillin T, Hughes AD, Wang Q, Würtz P, Korpela MA, Sattar N, et al Diabetes risk and amino acid profiles: cross-sectional and prospective analyses of ethnicity, amino acids and diabetes in a South Asian and European cohort from the SABRE (Southall And Brent REvisited) Study. Diabetologia. 2015;58:968–79. 10.1007/s00125-015-3517-8 25693751 PMC4392114

[46] Yu H, Di J, Bao Y, Zhang P, Zhang L, Tu Y, et al Visceral fat area as a new predictor of short-term diabetes remission after Roux-en-Y gastric bypass surgery in Chinese patients with a body mass index less than 35 kg/m2. Surg Obes Relat Dis. 2015;11:6–13. 10.1016/j.soard.2014.06.019 25547054

[47] Ma RC, Chan JC. Type 2 diabetes in East Asians: similarities and differences with populations in Europe and the United States. Ann Ny Acad Sci. 2013;(1281):64–91. 10.1111/nyas.12098 PMC3708105 23551121

[48] Cho YS, Chen CH, Hu C, Long J, Ong RT, Sim X, et al Meta-analysis of genome-wide association studies identifies eight new loci for type 2 diabetes in east Asians. Nat Genet. 2012;44(1):67–72. 10.1038/ng.1019 PMC3582398 22158537

[49] Fernstrom JD. Large neutral amino acids: dietary effects on brain neurochemistry and function. Amino Acids. 2013;45(3):419–30. 10.1007/s00726-012-1330-y . 22677921

