# Supplementary material for: Tryptophan Predicts the Risk for Future Type 2 Diabetes
Source: PLoS One. 2016 Sep 6;11(9):e0162192. doi: 10.1371/journal.pone.0162192 (PMC5012675; doi:10.1371/journal.pone.0162192)
Supplement: S1 Fig — The r and p values were from Spearman correlation (n = 33). (DOCX) [file pone.0162192.s001.docx]

**S1 Fig. Scatter plot of T2D duration (year) and tryptophan level (ug/ml).**

The r and p values were from Spearman correlation (n=33)
