# Supplementary material for: Tryptophan Predicts the Risk for Future Type 2 Diabetes
Source: PLoS One. 2016 Sep 6;11(9):e0162192. doi: 10.1371/journal.pone.0162192 (PMC5012675; doi:10.1371/journal.pone.0162192)
Supplement: S1 Table — p1 was p values from Mann Whitney U test comparing T2D and NGT. FC (fold change) represents mean ratio of T2D to NGT. Crude odds ratio (Crude OR) and confidence interval (CI) per s.d., and p2 were from basic logistic regression models and S.D. scaled data. (DOCX) [file pone.0162192.s003.docx]

**S1 Table. Predictive performance of baseline amino acid ratios in discriminating individuals who developed diabetes in 10 years (T2D, n=51) from those who remained metabolically healthy (matched NGT, n=23).**

| Amino acid ratios | p1 | FC | Basic logistic model | |
| --- | --- | --- | --- | --- |
|  |  |  | Crude OR (95% CI) | p2 |
| Tryptophan /Valine | 0.38 | 1.09 |  |  |
| Tryptophan /Leucine | 0.70 | 1.07 |  |  |
| Tryptophan /Isoleucine | <0.01 | 0.61 | 0.93 (0.88, 0.98) | 0.005 |
| Tryptophan /Phenylalanine | 0.48 | 1.10 |  |  |
| Tryptophan /Tyrosine | <0.01 | 0.64 | 0.94 (0.88, 1.00) | 0.036 |

p1 was the p values from Mann Whitney U test comparing T2D and NGT.

FC (fold change) represents the mean ratio of T2D to NGT.

Crude odds ratio (Crude OR) and confidence interval (CI) per s.d., and p2 were from basic logistic regression models and S.D. scaled data.
